# Mapping the complement-targeted landscape in igA nephropathy: a systematic review with structured descriptive synthesis

**DOI:** 10.3389/fmed.2026.1933632

**Published:** 2026-10-02

**Authors:** Xin-Yu Li, Chang-Ying Zhong, Li-Nan Zhang, Yang Liu

**Affiliations:** 1 Institute of Rehabilitation and Health Care, Hunan Traditional Chinese Medical College, Hunan, China; 2 Otolaryngology Department, Hunan Provincial Hospital of Integrated Traditional Chinese and Western Medicine, Changsha, Hunan, China

**Keywords:** complement inhibitors, eGFR slope, IgA nephropathy, iptacopan, proteinuria, systematic review

## Abstract

**Background:**

Complement activation is a key driver of disease progression in IgA nephropathy (IgAN). While previous syntheses were limited to phase II evidence, pivotal phase III data—including the 24-month results of the APPLAUSE-IgAN trial—have recently emerged, necessitating an updated appraisal of complement-targeted therapies.

**Methods:**

We conducted a systematic literature search in PubMed, Web of Science, Embase, and Cochrane up to April 24, 2026, for randomized controlled trials of complement inhibitors in adults with biopsy-proven IgA nephropathy. Due to marked mechanistic heterogeneity across agents targeting distinct complement nodes, a quantitative meta-analysis was not performed. Instead, we performed a structured descriptive synthesis, a predefined, domain-based comparative method, focusing on efficacy signals, safety profiles, and methodological disparities across trials.

**Results:**

Four RCTs comprising 584 patients were included, representing factor B and C5 inhibition. The phase III iptacopan trial demonstrated robust proteinuria reduction, eGFR slope attenuation, and a decreased risk of kidney failure, but a higher serious-infection rate and confirmed pneumococcal infections highlighted that alternative-pathway inhibition attenuates rather than abolishes encapsulated-bacteria risk. Phase II trials of ravulizumab, iptacopan, and cemdisiran provided early efficacy signals with no encapsulated-bacterial events reported, though small size and short follow-up limit safety inference. All trials required prophylactic vaccination, and infection-risk obligations differed by target—terminal-pathway inhibitors carrying the strictest REMS requirements. Methodological heterogeneity—particularly regarding biopsy timing and fibrosis exclusion—emerged as a major barrier to cross-trial comparisons. Beyond these RCTs, a dynamic pipeline of ASO, siRNA, and small-molecule inhibitors continues to evolve.

**Conclusion:**

Complement inhibitors show promising but heterogeneous signals across individual trials; however, marked differences in molecular targets, trial design, and eligibility preclude any inference of a uniform class effect. Infection risk mitigation differs by complement target—terminal-pathway inhibitors carry the highest encapsulated-bacterial burden and strictest regulatory constraints, whereas alternative-pathway inhibitors preserve partial vaccine-mediated defence but still require comprehensive vaccination and surveillance. These observations are hypothesis-generating and underscore the need for mechanism-specific phase III programmes with hard renal endpoints and individual patient-level meta-analyses to advance precision complement therapeutics in IgAN.

**Systematic Review Registration:**

https://www.crd.york.ac.uk/PROSPERO/view/CRD420261385690

## 1 Introduction

IgA nephropathy (IgAN) is a chronic kidney disease characterized primarily by recurrent episodes of gross hematuria and proteinuria (1). While the global incidence of IgAN is approximately 2.5 per 100,000 individuals, it constitutes a significantly higher proportion of biopsy-proven glomerulonephritis in East Asian countries, including China, Japan, and South Korea, ranging from 27% to 51.6% (2). Recent investigations have increasingly implicated dysregulated immune responses and subsequent immune complex deposition in the pathogenesis of IgAN, with the ‘four-hit' hypothesis currently being the most widely accepted mechanism. Its pathogenesis is complex and remains controversial. The primary focus of the controversy lies in the unclear origin of galactose-deficient IgA1 (Gd-IgA1) in blood (3, 4). Guided by the prevailing pathophysiological theories, numerous novel therapeutics are under development. A prominent example is complement inhibitor. Complement activation has been recognized as a key driver of kidney injury in IgA nephropathy (5), particularly via the alternative and terminal pathways. In recent years, there has been significant progress in understanding the pathogenic mechanisms of complement activation (6–8). While no validated complement biomarkers currently guide treatment selection, complement inhibitors targeting factor B (FB), C5, and C3 have emerged as promising therapeutic options (9). Surprisingly, to date, randomized controlled trials (RCTs) targeting other arms of the complement pathway have yet to demonstrate efficacy (10). While a systematic review of complement-targeted therapies for IgAN was published, its findings were derived exclusively from phase II trials (11). Recently, pivotal phase III data—specifically the full 24-month results of the APPLAUSE-IgAN study—have become available, necessitating an updated synthesis.

We did not perform a quantitative meta-analysis because the included trials target distinct complement nodes and differ substantially in phase, sample size, and endpoint definitions. We performed a structured descriptive synthesis (Section 2.2) to map the therapeutic landscape, focusing on efficacy signals, methodological heterogeneity, and implications for future trial design.

## 2 Material and method

### 2.1 Scope and evidence selection strategy

To capture the breadth of the field while maintaining a focus on high-quality evidence, we employed a systematic search strategy across PubMed, Web of Science, Embase, and the Cochrane Central Register of Controlled Trials (CENTRAL) from inception to June 24, 2026. Search terms integrated three conceptual domains: (1) disease entities (“IgA Nephropathy” OR “Berger's Disease”), (2) therapeutic targets (“complement inhibitor” OR “factor B inhibitor” OR “C3 inhibitor” OR “C5 inhibitor” OR “iptacopan” OR “cemdisiran” OR “ravulizumab”), and (3) study design (“randomized controlled trial” OR “phase II” OR “phase III”).

Given the nascent stage of clinical translation in this area, our inclusion criteria were deliberately broad yet rigorous: RCTs enrolling adults with biopsy-proven primary IgAN, evaluating any pharmacological modulator of the complement cascade (including monoclonal antibodies, small molecules, or RNA-based therapeutics), and reporting clinical outcomes [Urine Protein-to-Creatinine Ratio (UPCR) or estimated glomerular filtration rate (eGFR)]. To ensure methodological rigor, only RCTs in primary IgAN were included.

### 2.2 Analytical framework and synthesis approach

A quantitative meta-analysis was not performed for two predefined methodological reasons. First, the included trials target distinct nodes of the complement cascade, violating the intervention-similarity assumption required for random-effects pooling. Second, the planned synthesis anticipated a mixed-phase evidence base with sparse phase III data and predominantly small phase II trials, making pooled effect estimates unstable and difficult to interpret.

Instead, we conducted a structured descriptive synthesis, defined here as a predefined, domain-based comparative method in which each trial is extracted into a standardized template covering: (i) mechanistic target; (ii) trial phase and sample size; (iii) eligibility and histopathological criteria; (iv) efficacy outcomes (UPCR reduction, eGFR slope, hard renal endpoints); and (v) safety and tolerability. These data were then presented side-by-side in Tables 1–4 to facilitate cross-trial comparison without generating pooled effect estimates.

**Table 1:** Individual trial effects on UPCR reduction.

Study	Complement Target	Phase	GMR (95% CI)	% Reduction vs. Placebo	n (Exp)	n (Ctrl)
Barratt et al. 2026 (Iptacopan)	Factor B (alternative pathway)	III	0.61 (0.54–0.69)	39%	226	211
Lafayette et al. 2025 (Ravulizumab)	C5 (terminal pathway)	II	0.67 (0.52–0.85)	33%	43	23
Barratt et al. 2024 (Cemdisiran)	C5 (terminal pathway)	II	0.69 (0.45–1.06)	31%	22	8
Zhang et al. 2024 (Iptacopan)	Factor B (alternative pathway)	II	0.77 (0.60–0.99)	23%	26	25

Exp, experimental (intervention) group; Ctrl, control (placebo) group; GMR, geometric mean ratio of UPCR; values <1 indicate reduction versus placebo. Data extracted from published trial reports.

**Table 2:** Individual trial effects on annualized eGFR slope.

Study	Complement Target	Phase	Delta eGFR slope (mL/min/1.73m^2^/yr)	95% CI	n (Exp/Ctrl)
Barratt et al. 2026 (Iptacopan)	Factor B	III	+3.02	2.12 to 4.08	226/211
Lafayette et al. 2025 (Ravulizumab)	C5	II	+5.39	0.04 to 10.52	43/23
Barratt et al. 2024 (Cemdisiran)	C5	II	+5.14	0.02 to 10.26	22/8

Positive values indicate slower eGFR decline versus placebo. The phase II Iptacopan trial did not report eGFR slope and was excluded. Wide CIs in phase II trials reflect small sample sizes.

**Table 3:** Treatment-emergent AE summary.

Study	Phase	Target	AE: n (Exp)	AE: n (Ctrl)	RR (95% CI)
Barratt et al. 2026 (Iptacopan)	III	FB	207 (238)	213 (239)	0.98 (0.91–1.04)
Lafayette et al. 2025 (Ravulizumab)	II	C5	32 (43)	19 (23)	0.90 (0.70–1.16)
Barratt et al. 2024 (Cemdisiran)	II	C5	19 (22)	8 (8)	0.87 (0.74–1.02)
Zhang et al. 2024 (Iptacopan)	II	FB	14 (26)	18 (25)	0.75 (0.49–1.05)

**Table 4:** Summary of key methodological characteristics and eligibility criteria across included studies.

Comparison Dimension	Iptacopan phase III	Ravulizumab phase II	Cemdisiran phase II	Iptacopan phase II
Age Range	=> 18 years	18–75 years	18–65 years	18–75 years
Biopsy Requirements	Stratified: eGFR =>45 (within 5y); 30–45 (within 2y & fibrosis <50%)	Any time (pre/post screening); Main cohort eGFR =>30	Within 60 months prior to screening	Pre-screening or during screening; no time restriction
eGFR Lower Limit	=> 30 mL/min/1.73m^2^ (with 20–30 exploratory cohort)	=> 30 mL/min/1.73m^2^ (with 20-<30 exploratory cohort)	=> 30 mL/min/1.73m^2^	=> 30 mL/min/1.73m^2^
Proteinuria Threshold	24h-UPCR => 1.0 g/g (on stable RASi => 90 days)	24h-UP => 1.0 g or UPCR => 0.75 g/g (on stable RASi >= 3 months)	24h-UP => 1.0 g (on stable RASi => 3 months)	24h-UPCR => 1.0 g/g (on stable RASi => 90 days)
Hematuria Requirement	NR	NR	Required (Microscopy => 10 RBC/HPF or Dipstick = > 1+)	NR
Immunosuppression Exclusion	Prednisone > 20 mg/d or IS > 6 months	Systemic steroids/IS > 6 months	Steroids/IS > 6 months	Prednisone > 20 mg/d or IS > 6 months
Vaccination Requirement	Meningococcal, Pneumococcal, Influenza (Hib recommended); prophylactic antibiotics if unvaccinated	Meningococcal (within 3 years); prophylactic antibiotics if unvaccinated	Meningococcal, Pneumococcal, Hib (=> 4 weeks prior); prophylactic antibiotics as needed	Meningococcal (within 3 years); prophylactic antibiotics if unvaccinated
SGLT2i Requirement	Allowed (stable dose)	Allowed (stable => 3 months, no planned changes)	Allowed (stable RASi => 3 months, BP controlled)	Allowed (stable => 3 months)
Primary Endpoint Time	9 months	6.5 months	8 months	3 months
Maximum Follow-up	24 months	12.5 months	36 months	6 months

NR, not reported; Primary endpoint was UPCR reduction at the specified time point for all trials.

This approach differs from a free-text narrative review by fixing the extraction schema *a priori* and by systematically appraising methodological heterogeneity. It differs from meta-analysis by deliberately avoiding quantitative aggregation across mechanistically distinct agents. The method aligns with guidance for narrative and scoping syntheses where pooling is inappropriate (McKenzie and Brennan, Cochrane Handbook Ch.12, v6.5) (12). Risk of bias for each trial was assessed qualitatively using the Cochrane RoB 2 tool and integrated into the interpretation of trial findings.

## 3 Result

### 3.1 Literature search and study selection

A total of 510 articles were initially identified through database and register searches. After removing 14 duplicate records, 496 unique articles remained for screening. Based on predefined criteria, 442 articles were excluded at the title/abstract screening stage for reasons shown in Figure 1. Additionally, 35 overlapping studies were excluded in the identification, leaving 19 articles eligible for full-text retrieval. Of these, 15 articles were excluded. Four studies met all inclusion criteria and were included in the final narrative synthesis. These four studies were Barratt et al. 2026 (13), Zhang et al. (14), Barratt et al. 2024 (15), Richard Lafayette et al. 2025 (16).

**Figure 1:**
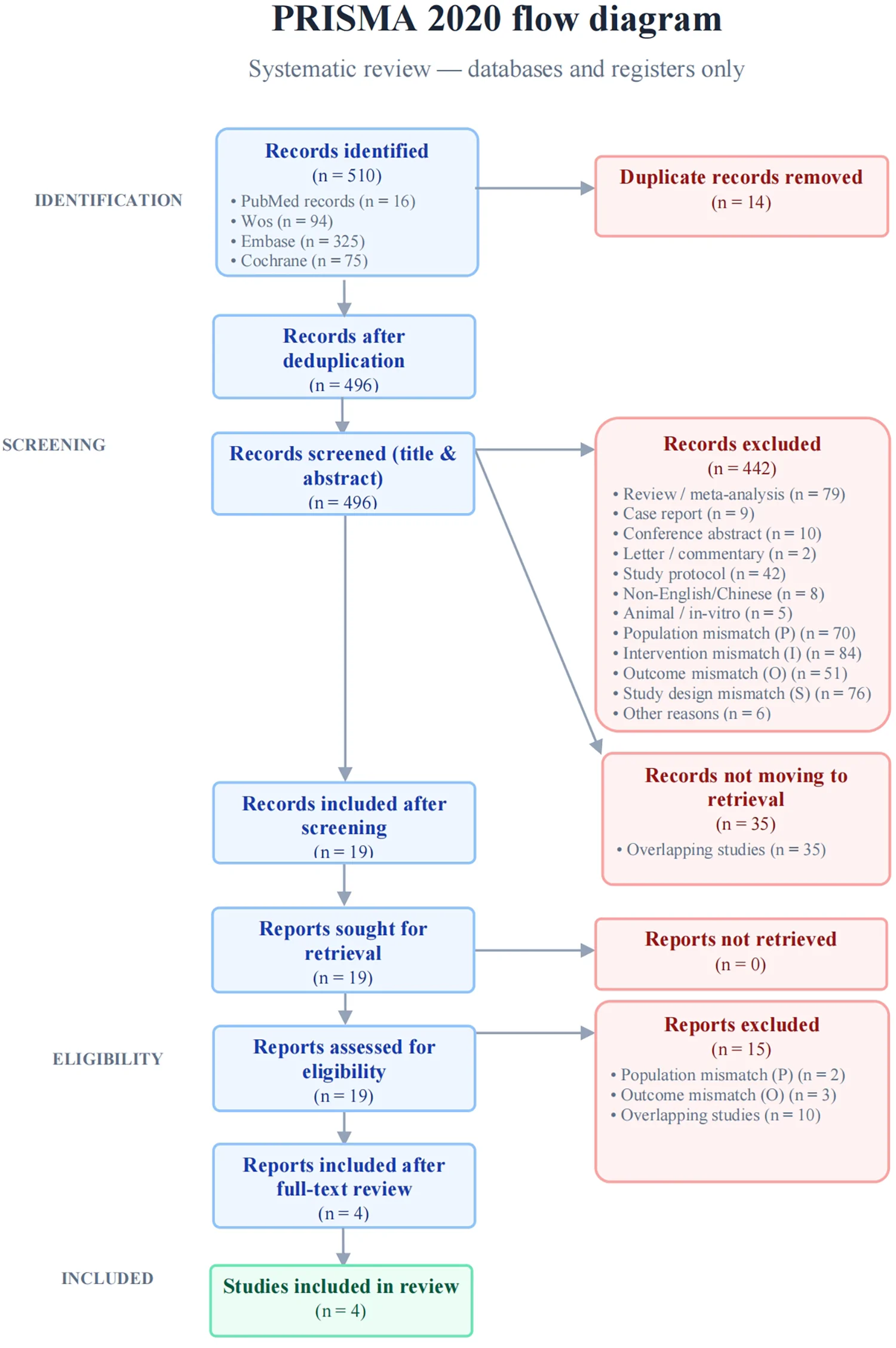
PRISMA 2020 flow diagram of study selection for complement inhibitors in IgAN.

In Figure 2, overall, all four included trials were judged to have “some concerns” regarding bias based on the RoB 2.0 assessment. Specifically, all trials raised concerns in the domains of Selective Reporting and Randomization, while the Outcome Measurement and Deviations domains were uniformly rated as low risk, reflecting standardized and reliable assessment of UPCR and eGFR across studies.

**Figure 2:**
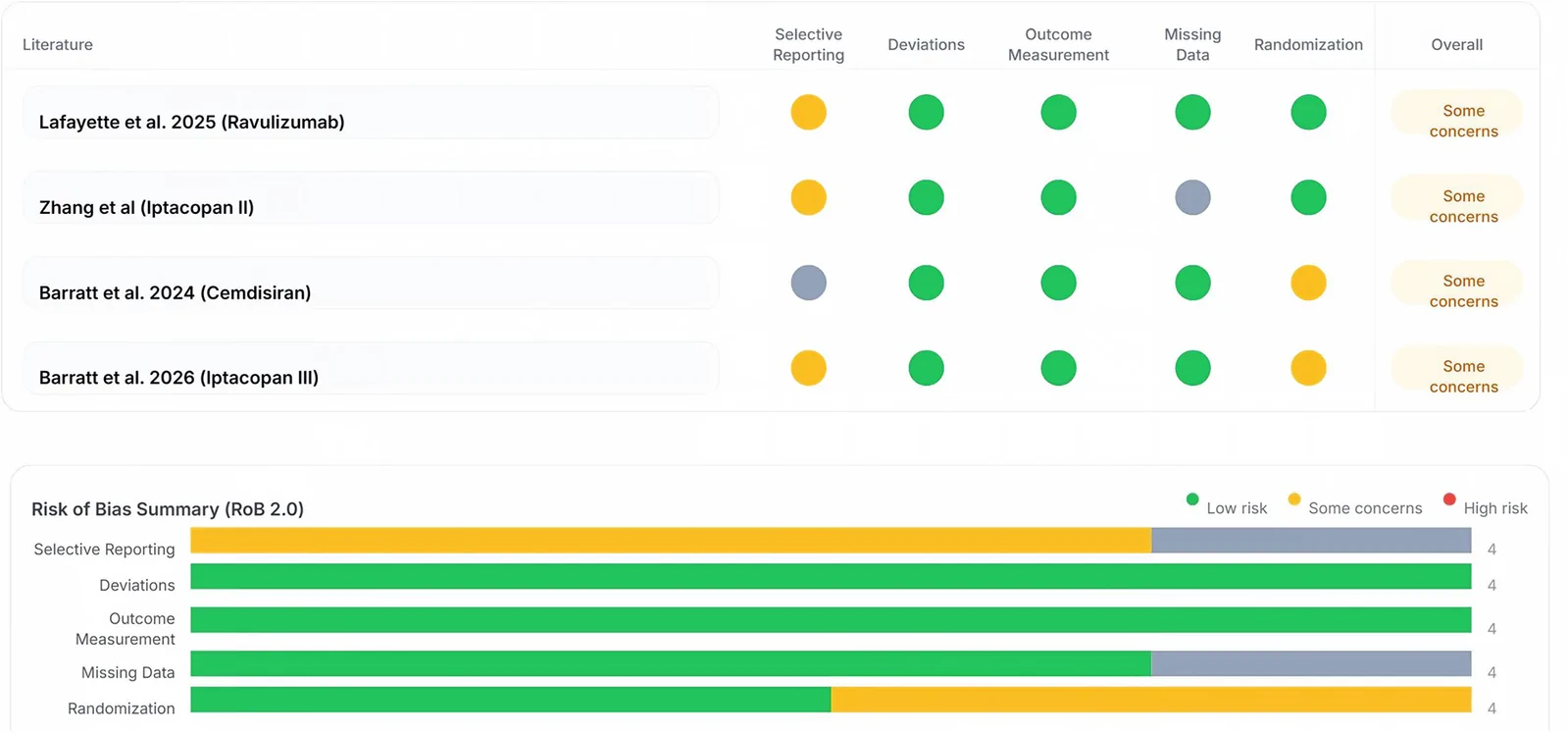
Summary of risk of bias.

### 3.2 Landscape of clinical evidence for complement inhibitors

Our systematic search identified four pivotal RCTs comprising 584 patients, representing the totality of high-level evidence currently available. These trials delineate two distinct mechanistic classes of complement inhibitors: (1) factor B inhibitors targeting the alternative pathway (iptacopan, phase II and III); (2) a C5 inhibitor targeting the terminal pathway (ravulizumab, phase II); and another terminal pathway C5 inhibitor (cemdisiran, phase II).

A critical limitation of the current evidence base is the pronounced asymmetry in developmental maturity across complement inhibitors. The phase III APPLAUSE-IgAN trial of iptacopan is the largest and only phase III contribution among the four included studies, enrolling substantially more participants than the three phase II trials combined and thereby anchoring the current evidence base. In contrast, the remaining agents are confined to the phase II setting, typically involving smaller sample sizes (<70 patients) and different follow-up durations. This developmental disparity creates a hierarchical structure in the evidence base, where conclusions regarding class-wide efficacy must be tempered by the recognition that current perceptions are heavily influenced by the performance of a single agent.

### 3.3 Efficacy signals across mechanistic classes

As shown in Tables 1, 2, factor B inhibition (Alternative Pathway): The phase III trial of iptacopan established a new benchmark for efficacy. Over 24 months, iptacopan demonstrated a robust 39% reduction in UPCR [Geometric Mean Ratio (GMR) 0.61] and a clinically meaningful 3.02 mL/min/1.73m^2^/year slowing of eGFR decline relative to placebo. Crucially, this trial provided the hard endpoint data in this therapeutic class, showing a 43% relative risk reduction in a composite kidney failure outcome (HR 0.57, 95% CI 0.40–0.81). A prior phase II trial of iptacopan showed a more modest 23% UPCR reduction at 3 months, potentially reflecting shorter exposure and different baseline characteristics.

Terminal Pathway Inhibition (C5): The phase II trial of ravulizumab reported a 33% UPCR reduction (GMR 0.67). While the eGFR slope improved by 5.39 mL/min/1.73m^2^/year, the clinical significance of this finding is limited by the short follow-up and the inherent variability of slope estimates in small populations. The other phase II trial of cemdisiran demonstrated a 31% UPCR reduction (GMR 0.69) at 8 months. Similar to ravulizumab, the reported eGFR slope benefit (5.14 mL/min/1.73m^2^/year) requires validation in larger, longer-duration trials to confirm renal protection beyond proteinuria lowering.

### 3.4 Safety profiles and tolerability

Across the four trials, complement inhibitors exhibited a generally favorable and consistent safety profile across mechanistic classes, with no unexpected toxicities emerging in Table 3. In the phase III APPLAUSE-IgAN trial, the incidence of treatment-emergent adverse events (TEAEs) was 207 (238) in the phase III iptacopan arm versus 213 (239) in the placebo arm (RR 0.98, 95% CI 0.91–1.04). Phase II trials similarly reported no significant increase in overall AE risk in the intervention arms: ravulizumab 32/43 versus 19/23 (RR 0.90, 95% CI 0.70–1.16), cemdisiran 19/22 versus 8/8 (RR 0.87, 95% CI 0.74–1.02), and phase II iptacopan 14/26 versus 18/25 (RR 0.75, 95% CI 0.49–1.05).

Regarding encapsulated bacterial infections, all four trials required prophylactic vaccination per protocol. Only the phase III iptacopan trial reported such events: 10 (4.2%) in the experimental arm versus 8 (3.3%) in the placebo arm, including two confirmed pneumococcal infections. Overall, serious infections occurred in 16 patients (6.7%) receiving iptacopan and in 5 (2.1%) receiving placebo. The phase II trials of ravulizumab, cemdisiran, and iptacopan reported no encapsulated bacterial infections. The safety dataset is dominated by the phase III iptacopan trial. The specific vaccine requirements are documented in Supplementary Table S2.

### 3.5 Methodological heterogeneity: a barrier to cross-trial inference

Perhaps the most significant finding of this review—and a major impediment to quantitative synthesis—is the profound methodological heterogeneity across trials (Table 4). Key discrepancies include:

Histopathological Entry Criteria: The phase III iptacopan trial mandated a recent biopsy (within 5 years for eGFR =>45 mL/min/1.73m^2^ or 2 years for eGFR 30–45 mL/min/1.73m^2^) and strictly excluded patients with >50% interstitial fibrosis. Conversely, phase II trials often relied on older biopsies or clinical diagnoses, potentially enrolling a more fibrotic, less treatment-responsive phenotype.

Baseline Proteinuria Thresholds: While most trials required a UPCR =>1.0 g/g, the ravulizumab trial utilized a lower threshold (=>0.75 g/g), potentially capturing a different natural history cohort.

These methodological divergences imply that apparent differences in efficacy between agents may reflect differences in enrolled populations rather than true pharmacological variation.

### 3.6 Other studies not included but that are ongoing or have been discontinued

Within the landscape of complement-targeted therapies for IgAN, agents across different technological platforms exhibit varying developmental trajectories and efficacy signals.

In the antisense oligonucleotide (ASO) domain, Sefaxersen (formerly IONIS-FB-LRx) demonstrated a mean 43% reduction in UPCR at 29 weeks in a Phase II single-arm study enrolling 23 patients (17). This compound has since advanced to the pivotal Phase III IMAGINATION trial. Regarding small-molecule inhibitors, the FB inhibitor HSK39297 was evaluated in a randomized, double-blind, Phase II study (conducted in China) involving 87 patients. At 12 weeks, the treatment group achieved a UPCR reduction of at least 48.2%, significantly outperforming the 5.3% reduction observed in the placebo group (18). Another FB inhibitor, HRS-5965, remains in the clinical trial phase; while early data suggest significant complement inhibition, comprehensive efficacy results are pending, and the study population is not restricted to IgAN (19).

Additionally, the C5a receptor (C5aR) inhibitor avacopan was assessed in a small Phase II pilot study (*n* = 7). Short-term (12-week) data demonstrated a mean UPCR reduction of approximately 50%, with responders observed in 3 of 7 patients; however, long-term efficacy and safety data remain lacking (20). Although long-term follow-up data are lacking, a Phase II trial targeting crescentic IgAN (NCT06676579) is currently recruiting. In the realm of RNA interference (RNAi) and peptide therapeutics, the C3-targeted small interfering RNA (siRNA) agent ARO-C3 is in early-stage exploration. In its Phase IIa study, a 41.1% mean reduction in spot UPCR was observed after 24 weeks of treatment in 14 patients (21). Conversely, pegcetacoplan—a C3-targeted peptide approved for C3 glomerulopathy—remains in the exploratory stage for IgAN. Its Phase II study included 23 patients (6 diagnosed with IgAN); however, findings to date have focused primarily on safety, yielding conservative conclusions (22). Finally, in the monoclonal antibody space, the lectin pathway inhibitor narsoplimab (targeting MASP-2) progressed to Phase III but was terminated following the ARTEMIS-IGAN trial (NCT03608033). Interim analysis revealed that narsoplimab failed to significantly reduce proteinuria compared to placebo (23). The results were summarized in Table 5.

**Table 5:** Characteristics of other complement inhibitors in IgAN.

Drug Name (Type)	Pathway	Specific Target	Development Stage (IgAN Indication)	Key Efficacy Signal (vs. Placebo)
Sefaxersen (ASO)	Alternative Pathway	Complement Factor B (FB) mRNA	Phase III (IMAGINATION)	Phase II: 43% UPCR reduction at 29 weeks
HSK39297 (Small molecule)	Alternative Pathway	Complement Factor B (FB)	Phase II (China)	Phase II preliminary: > 48.2% UPCR reduction at 12 weeks
HRS-5965 (Small molecule)	Alternative Pathway	Complement Factor B (FB)	Phase II (China)	Early clinical data show significant complement inhibition
Pegcetacoplan (Peptide)	Central/Amplification	Complement C3	Phase II	Early proof-of-concept shows trend toward proteinuria reduction
ARO-C3 (siRNA)	Central/Amplification	Complement C3	Phase I/IIa	Early data show significant complement inhibition; large-scale efficacy data pending
Avacopan (Small molecule)	Inflammatory Effector	C5a Receptor (C5aR)	Phase II	Open-label Phase II: 50% UPCR improvement at 12 weeks (partial)
Narsoplimab (mAb)	Lectin Pathway	MASP-2	Phase III (Terminated)	Failed: ARTEMIS-IGAN Phase III missed primary endpoint

mAb, monoclonal antibody; ASO, antisense oligonucleotide; siRNA, small interfering RNA; FB, Factor B; C5, complement C5; C3, complement C3; C5aR, C5a receptor; MASP-2, MBL-associated serine protease 2.

## 4 Discussion

### 4.1 Principal findings

This structured descriptive synthesis integrates the first phase III evidence with existing phase II data on complement inhibitors in IgA nephropathy. Across the four included RCTs, directional improvements in surrogate renal endpoints were consistently observed. The phase III APPLAUSE-IgAN trial established a new benchmark, demonstrating both a significant reduction in proteinuria and a meaningful slowing of eGFR decline, alongside a reduction in hard renal endpoints. In the subcohort of the same trial of iptacopan for patients with eGFR <30 mL/min/1.73 m^2^, the median UPCR demonstrated a relative reduction of 34.5% at nine months compared to the control group (24). In contrast, phase II trials of ravulizumab and cemdisiran provided early signals of efficacy, albeit with shorter follow-up and smaller sample sizes. Importantly, these findings should not be interpreted as evidence of a uniform class effect. The diversity in molecular targets among the included trials—spanning the alternative pathway (factor B) and the terminal complement cascade (C5)—suggests that treatment effects are likely drug- and mechanism-specific rather than attributable to a uniform complement-class effect. Consequently, the observations herein are best viewed as hypothesis-generating, underscoring the need for mechanism-specific evaluations in future research.

### 4.2 Interpretation of evidence across trials

A critical insight from this review is the substantial methodological heterogeneity among included studies. The phase III iptacopan trial employed stringent eligibility criteria, including mandatory recent biopsy and exclusion of patients with extensive interstitial fibrosis, thereby selecting for a more homogeneous and potentially treatment-responsive population. Conversely, phase II trials often allowed older histopathological diagnoses. These differences in enrollment practices, combined with variations in background therapy standardization and endpoint timing, imply that apparent differences in efficacy may reflect population selection rather than true pharmacological divergence. Without individual patient-level data, disentangling these influences remains infeasible. Furthermore, the current evidence base is heavily weighted toward a single agent (iptacopan); thus, caution is warranted when extrapolating these findings to other complement inhibitors.

### 4.3 Infection risk mitigation across complement targets in igA nephropathy

All four trials mandated meningococcal vaccination at enrolment, with antibiotic prophylaxis if not up to date; pneumococcal and Hib vaccination was recommended per protocol or local guidelines. Notably, only the iptacopan studies required all three vaccinations, whereas the C5-directed trials specified meningococcal coverage only. Only the phase III iptacopan trial reported encapsulated-bacterial events; the phase II trials reported none, reflecting small size and short follow-up rather than confirmed safety. Terminal complement deficiency predisposes to meningococcal infection because membrane attack complex is required for serum bactericidal killing of N. meningitidis (25). C5 inhibitors reproduce this defect; labels for ravulizumab therefore mandate vaccination and Risk Evaluation and Mitigation Strategy (REMS) enrolment, explicitly stating that vaccination does not eliminate encapsulated-bacterial risk (26). Mechanistically, C5 inhibition abolishes meningococcal serum bactericidal activity even in vaccinated sera, whereas factor B inhibition impairs killing only at low anti-capsular antibody titres, as classical-pathway activation remains intact (27).

Consensus guidance accordingly treats complement inhibition as a spectrum: MenACWY (meningococcal serogroups A, C, W, and Y) and MenB (meningococcal serogroup B) are required for all complement inhibitors, while pneumococcal/Hib coverage, antibiotic prophylaxis and fever-action plans are recommended by pathway and indication (28). A nephrology-focused practical review confirms that eculizumab, ravulizumab, iptacopan and pegcetacoplan all require REMS, whereas avacopan does not spare C5b-9 formation; alternative-pathway inhibitors may preserve vaccine-driven classical-pathway killing better than terminal-pathway inhibitors, though long-term real-world data remain limited (29). In PNH, 48-week iptacopan data showed no new serious encapsulated-bacterial infections during the extension, although two occurred in the core period and longer follow-up is needed (30). In IgA nephropathy, APPLAUSE-IgAN reported no meningococcal or Hib infections over 24 months but confirmed two serious pneumococcal infections (6.7% vs. 2.1% serious infections overall), demonstrating that factor B inhibition attenuates—yet does not eliminate—encapsulated-bacteria risk, and that the dominant pathogen may vary by indication and population. *In vitro*, C3 and C5 inhibition abrogated Hib serum bactericidal activity regardless of vaccination, while factor B inhibition impaired killing only in vaccine-naïve serum, supporting a narrower defence gap for alternative pathway inhibitors when patients are appropriately immunized (31).

In short, complement-inhibitor infection risk is pathway-and target-dependent: terminal-pathway blockade carries the highest encapsulated-bacterial burden and strictest regulatory constraints, whereas alternative-pathway inhibitors preserve partial vaccine-mediated defence but still require comprehensive vaccination, surveillance, and REMS enrolment.

### 4.4 Surrogate endpoints and clinical relevance

Current evidence remains constrained by reliance on surrogate endpoints. Only the phase III iptacopan trial prospectively assessed a composite kidney failure outcome, providing a direct link between complement inhibition and clinically meaningful disease modification. The remaining phase II trials reported UPCR and eGFR slope as primary outcomes, which, while predictive of long-term outcomes, do not substitute for hard renal endpoints. Additionally, the long-term safety profiles of C5 inhibitors require further characterization in larger, more diverse patient populations. These limitations highlight the necessity of awaiting additional phase III data before drawing definitive conclusions regarding routine clinical adoption.

### 4.5 Pipeline developments and future directions

Definitive conclusions regarding the routine clinical adoption of complement inhibitors must await further phase III evidence with extended follow-up and validated hard renal endpoints. Future syntheses should prioritize mechanism-specific or agent-specific appraisals rather than pooling across the heterogeneous complement inhibitor class. Notably, individual patient-level data approaches will be essential to disentangle drug-specific effects from those attributable to shared pathway inhibition, thereby facilitating the advancement of precision complement therapeutics in IgA nephropathy. Accordingly, upcoming phase III trials represent key milestones for the field. These include the ongoing multicenter evaluations of ravulizumab (32, 33), as well as anticipated results for agents such as sefaxersen (17) and HSK39297 (18).

## 5 Conclusion

This structured descriptive synthesis maps the emerging complement-targeted landscape in IgA nephropathy by integrating the first phase III dataset with earlier phase II trials. The four included RCTs target distinct nodes of the complement cascade and differ substantially in design, eligibility, and endpoints, precluding meta-analysis and any inference of a uniform class effect. While directional improvements in proteinuria and eGFR trajectory were observed across individual trials, safety profiles require pathway-specific interpretation: factor B inhibition attenuates but does not eliminate encapsulated-bacteria risk, whereas terminal-pathway blockade carries the highest encapsulated-bacterial burden and strictest regulatory constraints. These findings underscore that comprehensive vaccination, surveillance, and REMS enrolment remain mandatory across complement targets. Stringent enrollment criteria limit the generalizability of these findings to real-world populations with advanced fibrosis or comorbidities. Definitive conclusions regarding routine clinical adoption must await further phase III data with hard renal endpoints and extended follow-up. Future progress requires mechanism-specific evaluation, predictive biomarker identification, standardized infection-risk reporting, and individual patient-level meta-analyses to advance precision complement therapeutics in IgA nephropathy.

## Data Availability

The original contributions presented in the study are included in the article/Supplementary Material, further inquiries can be directed to the corresponding author.
