# Supplementary material for: Mapping the complement-targeted landscape in igA nephropathy: a systematic review with structured descriptive synthesis
Source: Front Med (Lausanne). 2026 Oct 2;13:1933632. doi: 10.3389/fmed.2026.1933632 (PMC13671920; doi:10.3389/fmed.2026.1933632)
Supplement: Supplementary file 1 [file Table1.docx]

**Supplementary Table 1 Data Extraction of four studies**

| **Study** | **Barratt et al. 2026**  **(Iptacopan III)** | **Lafayette et al. 2025**  **(Ravulizumab phase II)** | **Barratt et al. 2024**  **(Cemdisiran phase II)** | **Zhang et al. 2024**  **(Iptacopan**  **phase II)** |
| --- | --- | --- | --- | --- |
| **phase** | III | II | II | II |
| **Drug** | Iptacopan | Ravulizumab | Cemdisiran | Iptacopan |
| **N (int/ctl) ^1^** | 238/239 | 43/23 | 22/9 | 26/25 |
| **Age(int/ctl)^2^** | 39.7±11.6/39.2±12.5 | 41±10/39±12 | 41±10/38±10 | 42.5±15.8/  39.4±11.0 |
| **Male%(int/ctl)** | 134(56.3%)/121(50.6%) | 21(49)/15(65%) | 13(59%)/3(33%) | 15(58%)/  18(72%) |
| **Baseline**  **eGFR(int/ctl)**  **mL/min/1.73 m²** | 63.4±25.5/65.6±26.5 | 76±30/70±30 | 68(54-94)/47(39-76) | 57.9±28.9/  65.7±32.6 |
| **baseline UPCR(int/ctl)**  **(median,IQR)**  **(g/g)** | 1.7(1.3-2.4)/1.8(1.5-2.6) | 1.7±0.9/1.8±1.0 | 1.55±1.03/1.97±0.82 | 1.3±1.0/  1.3±0.6 |
| **Primary endpoint**  **GMR 24h UPCR at specific month**  **g/g** | 0.61 (9 month) | 0.668 (6.5 month) | 0.69 (8 month) | 0.77  (3 month) |
| **Hard endpoint** | Composite kidney-failure end point  HR: 0.57 (0.40-81) | NR | NR | NR |
| **Max FU** | 24 months | 12.5 months | 36 months | 6 month |
| **Secondary endpoint annualized eGFR slope** | annualized eGFR slope estimate over 24 months | annualized eGFR slope estimate over 4.5 months | annualized eGFR slope estimate over 8 months | UPCR at 3 months |
| **delta eGFR slope** | 3.02 | 5.39 | 5.14 | NR^4^ |
| **AE(int/ctl)^3^** | 207/213 | 32/19 | 19/8 | 14/18 |

**Supplementary Table 2 Vaccine requirement for four studies**

| **Study** | **Target** | **Meningococcal** | **Pneumococcal** | **Hib** | **Antibiotics** |
| --- | --- | --- | --- | --- | --- |
| Zhang 2024 | FB | required, =>4 weeks prior | required, =>4 weeks prior | required, =>4 weeks prior | At investigator discretion |
| Barratt 2026 | FB | required | required | Recommended | — |
| Lafayette 2025 | C5 | required within 3 years | Not stated | Not stated | =>2 weeks post-vaccine |
| Barratt 2024 | C5 | required/willing | Per local guidelines | Per local guidelines | If required locally |
